# Supplementary material for: A Pipeline to Investigate Fungal–Fungal Interactions: Trichoderma Isolates against Plant-Associated Fungi
Source: J Fungi (Basel). 2023 Apr 10;9(4):461. doi: 10.3390/jof9040461 (PMC10142788; doi:10.3390/jof9040461)
Supplement: Supplementary file 1 [file jof-09-00461-s001.zip › jof-2223311-supplementary/Caption Videos.pdf]

A pipeline to investigate the interaction of soil fungi: nucleation dynamics of three *Trichoderma* strains against plant-associated fungi isolated from vineyards in Northern Italy

Marianna Dourou[a,b], Caterina A. M. La Porta[a,b,1,*]

[a] *Department of Environmental Science and Policy, University of Milan, Via Celoria 10, 20133 Milan, Italy*
[b] *Center for Complexity and Biosystems, University of Milan, Via Celoria 16, 20133 Milan, Italy*

Videos V1-V3: Time-lapse videos during single nucleation dynamic assay showing mycelium growth over time of the three *Trichoderma* strains. V1 refers to *Trichoderma simmonsii* EXF-17015 (No. 1); V2 refers to *Trichoderma* sp. EXF-17016 (No. 2); V3 refers to *Trichoderma* sp. EXF-17020 (No. 3).

Videos V4-V6: Time-lapse videos during dual nucleation dynamic assay showing mycelia growth over time of the three *Trichoderma* strains against themselves.
V4 refers to *Trichoderma simmonsii* EXF-17015 vs *Trichoderma simmonsii* EXF-17015; V5 refers to *Trichoderma* sp. EXF-17016 vs *Trichoderma* sp. EXF-17016; V6 refers to *Trichoderma* sp. EXF-17020 vs *Trichoderma* sp. EXF-17020.

Videos V7-V9: Time-lapse videos during dual nucleation dynamic assay showing mycelia growth over time of the three *Trichoderma* strains against the other *Trichoderma* strains. This behaviour is called mutual slight inhibition.
V7 refers to *Trichoderma simmonsii* EXF-17015 (No. 1) vs *Trichoderma* sp. EXF-17016 (No. 2); V8 refers to *Trichoderma* sp. EXF-17016 (No. 2) vs *Trichoderma* sp. EXF-17020 (No. 3); V9 refers to *Trichoderma simmonsii* EXF-17015 (No. 1) vs *Trichoderma* sp. EXF-17020 (No. 3).

*Corresponding author: Caterina A. M. La Porta, email: caterina.laporta@unimi.it

Video V10: Time-lapse video during the dual nucleation dynamic assay between *Trichoderma* sp. EXF-17020 (No. 3) vs *Rhizopus arrhizus* EXF-17019 (No. 8) over time. This behaviour is called mutually intermingling growth.

Video V11: Time-lapse video during the dual nucleation dynamic assay between *Trichoderma* sp. EXF-17020 (No. 3) vs *Fusarium remigenum* EXF-17018 (No. 7) over time. This behaviour is the subtype of the antagonistic called growth around.

Video V12: Time-lapse video during the dual nucleation dynamic assay between *Trichoderma* sp. EXF-17016 (No. 2) vs *Botrytis caroliniana* EXF-17025 (No. 9) over time. This behaviour is the subtype of the antagonistic called growth above.
