# Supplementary material for: A Pipeline to Investigate Fungal–Fungal Interactions: Trichoderma Isolates against Plant-Associated Fungi
Source: J Fungi (Basel). 2023 Apr 10;9(4):461. doi: 10.3390/jof9040461 (PMC10142788; doi:10.3390/jof9040461)
Supplement: Supplementary file 1 [file jof-09-00461-s001.zip › jof-2223311-supplementary/Fig. S1.pdf]

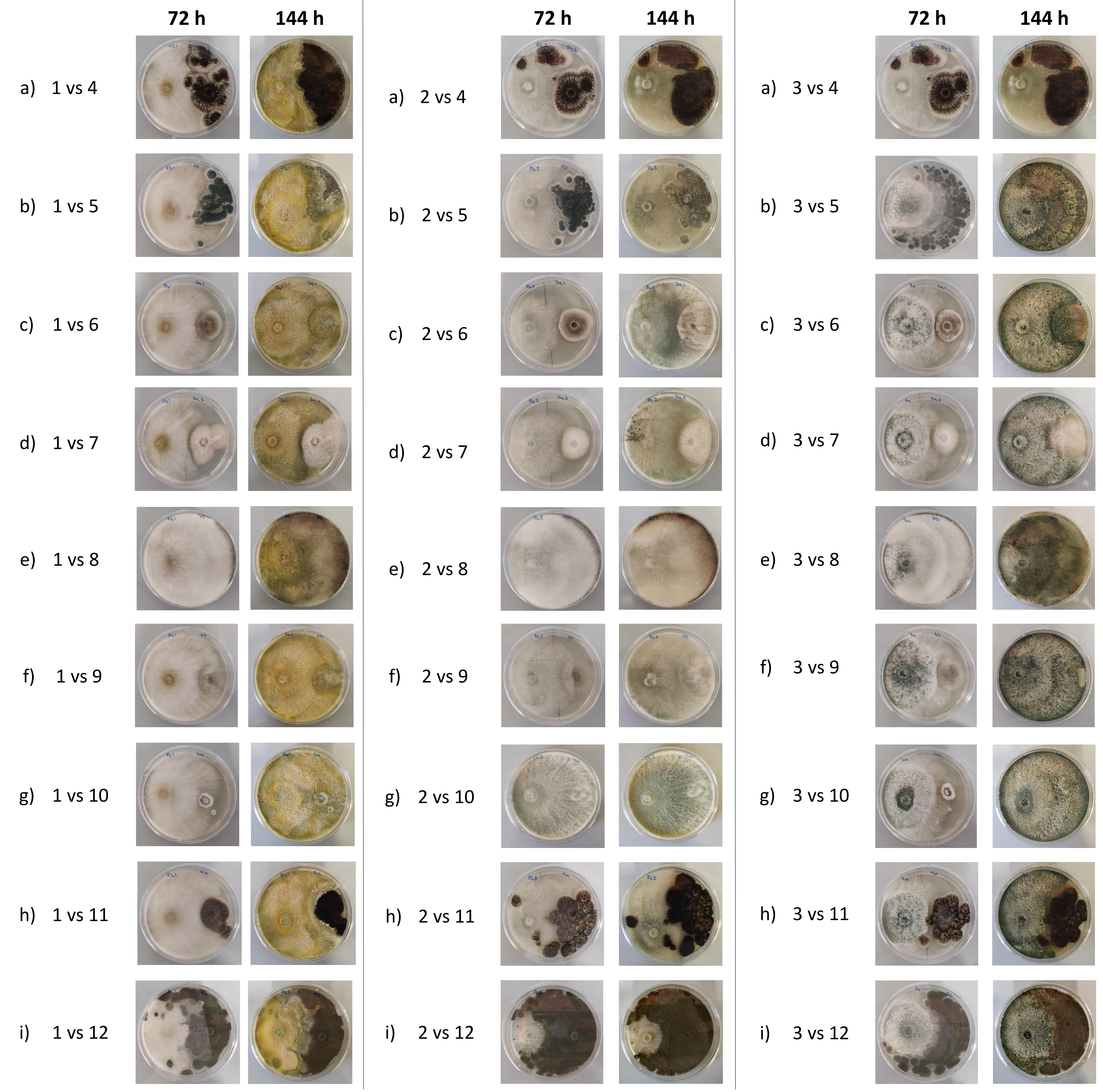

Figure S1. Dual nucleation dynamics of the three *Trichoderma* strains against the nine plant-associated fungi. Fungal cultures were performed in Petri dishes on a PDA medium. Pictures were taken 72 and 144 h post-cultivation.

Fungal strains: 1: *Trichoderma simmonsii* EXF-17015; **2:** *Trichoderma* sp. EXF-17016; **3:** *Trichoderma* sp. EXF-17020;
4: *Aspergillus aculeatus* EXF-17023; **5:** *Penicillium* sp. EXF-17021; **6:** *Alternaria* sp. EXF-17017;
7: *Fusarium ramigenum* EXF-17018; **8:** *Rhizopus arrhizus* EXF-17019; **9:** *Botrytis caroliniana* EXF-17025;
10: *Penicillium copticola* EXF-17026; **11:** *Aspergillus piperis* EXF-17024; **12:** *Penicillium oxalicum* EXF-17022
